# In-hospital growth and long-term neurodevelopmental outcomes of very low birth weight infants

**DOI:** 10.3389/fped.2023.1180068

**Published:** 2023-05-11

**Authors:** Alessandra Consales, Matteo Porro, Silvana Gangi, Nicola Pesenti, Laura Gardon, Chiara Squarza, Andrea Frigerio, Irene Lezzi, Giulia Vizzari, Daniela Morniroli, Marta Macchi, Camilla Fontana, Monica Fumagalli, Odoardo Picciolini, Fabio Mosca, Maria Lorella Giannì

**Affiliations:** ^1^ Department of Clinical Sciences and Community Health, University of Milan, Milan, Italy; ^2^ Fondazione IRCCS Ca' Granda Ospedale Maggiore Policlinico, Pediatric Physical Medicine & Rehabilitation Service, Milan, Italy; ^3^ NICU, Fondazione IRCCS Ca’ Granda Ospedale Maggiore Policlinico, Milan, Italy; ^4^ Division of Biostatistics, Epidemiology and Public Health, Department of Statistics and Quantitative Methods, University of Milano-Bicocca, Milan, Italy

**Keywords:** VLBW, growth, neurodevelopment, griffiths mental development scales, length, head circumference

## Abstract

**Background and Objectives:**

Very low birth weight infants (VLBW) are at risk for adverse growth and neurodevelopmental outcomes. We aimed to evaluate the association between growth during Neonatal Intensive Care Unit (NICU) stay and long-term neurodevelopmental outcomes in a cohort of preterm VLBW newborns.

**Methods:**

We conducted a longitudinal observational study in the Follow-up Service of our Clinic from January 2014 to April 2017. All preterm VLBW infants born at our hospital and enrolled in our follow-up program were considered eligible for the study. The neurodevelopmental assessment was performed using the Griffiths Mental Development Scales at 12 and 24 months corrected age.

**Results:**

Study population included 172 subjects (47.1% males) with a mean gestational age of 29 weeks and a mean birth weight of 1,117 g. A unitarian Δz-score increase in head circumference from birth to discharge was associated with a 1.6-point increase in General Quotient at 24 months corrected age. An association with subscales C and D was also found. Likewise, an increase in length Δz-score was associated with better 24-month subscale C scores although not reaching statistical significance. No relationship with the outcome at 24 months was found for weight gain.

**Conclusions:**

Growth during NICU stay appears to be related to a more favorable neurodevelopmental outcome at 24 months corrected age, especially in the hearing and language domain (subscale C). The longitudinal evaluation of auxological parameters during hospitalization can contribute to the identification of subjects at risk for adverse neurodevelopmental outcomes in the first years of life.

## 1. Background

Preterm infants are at increased risk for adverse neurodevelopmental outcomes secondary to the untimely exposure to extrauterine life and the development of gestational age-dependent comorbidities (1), such as sepsis (2), bronchopulmonary dysplasia (BPD) (3), necrotizing enterocolitis (NEC) (4), retinopathy of prematurity (ROP) (5), and severe brain lesions (SBL) (6). Although undeniably brain injuries occurring in the preterm infant affect the central nervous system development, prematurity *per se* is a major risk factor for impaired neurodevelopmental outcomes (7). Indeed, pivotal processes of brain growth and maturation occur between 24 and 40 weeks gestational age, including an increase in overall size and cortical folding (8). Disturbances in regional brain growth, alterations in white and grey matter microstructure as well as dysconnectivity of neural networks have been observed in prematurely born infants (9), even in the absence of overt brain lesions, and are implicated in the pathogenesis of adverse childhood outcomes, in particular in the cognitive domain, and in the risk of developmental psychiatric disorders (10). Growth failure has been shown to represent an additional risk factor for non-optimal neurological development in preterm infants (11). Indeed, among environmental factors that may act postnatally on a developing, therefore vulnerable, brain, nutritional insufficiencies play a key role (12, 13), although the impact of non-nutritional factors on growth trajectories should not be overlooked (14). Alongside quantity of growth, quality of growth should also be considered. Indeed, in preterm infants impaired growth is frequently associated with altered body composition development, which in turn may affect later metabolic and cognitive outcomes (15). Since in a clinical setting body composition assessment is not always feasible, length and head circumference may be used as reliable proxies to estimate lean mass accretion and brain growth, respectively, notwithstanding the importance of weight assessment, also considering the ease and reproducibility of its serial measurements.

Aim of the present study was to evaluate the association between growth (in terms of weight, length and head circumference) during the Neonatal Intensive Care Unit (NICU) stay and neurodevelopmental outcomes at 12 and 24 months corrected age (CA) in a cohort of preterm very low birth weight (VLBW) infants.

## 2. Materials and methods

### 2.1. Study design and setting

We conducted a monocentric longitudinal observational study in the Follow-up Service for newborns at risk of our center (Fondazione IRCCS Ca’ Granda Ospedale Maggiore Policlinico, Milan, Italy). Our hospital is a tertiary referral center for neonatal care, covering around 6,000 births per year. Our center offers an outpatient Follow-up service for prematurely born newborns, with low birth weight or relevant comorbidities, who have been discharged from the NICU.

This study is part of a larger project that investigated the neurological development of preterm VLBW infants admitted to the NICU of our centre from January 2014 to April 2017.

The study was approved by the Ethics Committee of the Fondazione IRCCS Ca' Granda Ospedale Maggiore Policlinico. Informed consent was obtained from both parents. All procedures were carried out according to the Declaration of Helsinki.

### 2.2. Study sample

All preterm infants (gestational age <37 weeks) with a birth weight <1,500 g consecutively born at our hospital from January 2014 to April 2017 and subsequently enrolled after discharge in the follow-up program routinely provided at our Institution were considered eligible for the study.

Infants affected by genetic syndromes and/or major congenital malformations, infants who died during NICU stay or after discharge, who were transferred to other hospitals before discharge, who abandoned the follow-up before reaching 24 months CA (drop-outs), whose data could not be retrieved or whose parents did not provide informed consent were excluded from the study.

### 2.3. Data collection

The baseline characteristics, perinatal history and follow-up information of the first 24 months CA of the infants enrolled were collected from the patients' computerized medical charts (Neocare i&t Informatica e Tecnologia Srl, Italy).

Basic socio-demographic characteristics were collected (i.e., maternal and paternal age, nationality and parental level of education).

Perinatal information collected included: gender, gestational age, mode of delivery, birth weight, length and head circumference, length of hospital stay, mode of feeding at discharge.

The mode of feeding was classified according to the WHO definitions (16).

The occurrence of common prematurity-associated pathologies was also collected.

During follow-up visits anthropometric measurements and neurodevelopmental assessment scores according to the Griffiths Mental Development Scale – Extended Revised (GMDS-ER) were recorded.

CA was calculated adjusting chronological age for gestational age.

Data were collected and analyzed anonymously.

### 2.4. Anthropometric measurements

During hospitalization and at follow-up visits infants' anthropometric parameters (weight, length and head circumference) were measured by registered pediatric nurses using standard techniques.

The weight was assessed with electronic scales, placing the naked infant on a light sheet. The measurement was approximated to the nearest gram.

The vertex-heel length was assessed with the Harpenden neonatometer. To guarantee the accuracy of the measurements, two health professionals were needed: one to hold the infant's head firmly against the headpiece, while keeping the shoulders fixed to the instrument, and the other one to fully extend the infant's right leg and hold the foot at a 90° angle to the sliding carriage. The measurements were approximated to the last completed millimeter.

The head circumference was measured by detecting the maximum occipito-frontal circumference using a non-extendable measuring tape placed on the frontal bones, above the supraorbital arches, and then passed around the head at the same height on both sides and posteriorly, passing through the most protruding point of the occiput. The measurements were approximated to the last completed millimeter.

During hospitalization the newborns' weight was assessed daily, whereas length and head circumference were assessed weekly, according to the hospital internal protocol.

### 2.5. Neurodevelopmental assessment

During follow-up visits the completion of the main stages of neuro-cognitive development was assessed by three trained and qualified examiners using the GMDS-ER. The Italian back-translation of the GMDS-ER was validated by Battaglia and Savoini in 2007 (17). The scale consists of 5 subscales, assessing different domains: Locomotor (A), Personal-Social (B), Hearing and Language (C), Eye and Hand Coordination (D) and Performance (E). Standardized General Quotient and Sub-quotients can be calculated. Typical development is expressed by a General Quotient ≥88 and a Sub-quotient ≥84 (i.e., within the first standard deviation—SD), whereas a General Quotient ≤87 and a Sub-quotient ≤83 are considered indicative of developmental delay (moderate if >1 SD below the mean and severe if >2 SD below the mean).

### 2.6. Statistical analysis

Demographic characteristics of the study population, growth parameters and neurodevelopmental outcomes at 12 and 24 months CA are presented using descriptive statistics. Continuous variables are expressed as mean and standard deviation or median and interquartile range; categorical variables are expressed as absolute and relative frequencies.

The z-scores for weight, length and head circumference at birth, during hospitalization and at discharge were assessed according to the Intergrowth-21st charts for Newborn Size for Very Preterm Infants and the longitudinal Charts for Post-Natal Growth of Preterm Infants (18, 19), respectively. Calculation of z-scores at 12 and 24 months CA was performed using the WHO Child Growth Standards (20).

To better evaluate the longitudinal growth of the subjects, the difference between the z-scores (Δz-score) of the anthropometric parameters (weight, length, head circumference) measured at discharge and those measured at birth was calculated. With regards to the weight, the Δz-score between its values from the time of weight recovery to discharge was also calculated.

A multivariate linear regression model was used to evaluate the association between auxological growth during hospitalization and neurodevelopmental outcome at 12 and 24 months CA as measured by the GMDS-ER scales (General Quotient and subscales A, B, C, D and E).

The model was corrected for the mode of feeding at discharge (human milk, formula milk and mixed feeding) and the Comorbidity Score. The Comorbidity Score is a severity score calculated for each newborn on the basis of their comorbidities, owing to their known impact on neurodevelopmental outcomes in VLBW infants (21). For each neurodevelopmental outcome studied, the single comorbidities are inserted in a linear model and the regression coefficients used as weights and added together, thus obtaining the score. The comorbidities included in the model are: BPD, sepsis, ROP, NEC and SBL.

Brain lesions were identified on both cranial ultrasound (cUS) and brain magnetic resonance imaging (MRI) performed according to our Institution's internal clinical imaging protocol that includes sequential cUS scans from birth to term equivalent age (TEA) and conventional brain MRI at TEA.

SBL were defined as: grade III intraventricular hemorrhage and/or parenchymal hemorrhagic venous infarction and/or posthemorrhagic ventricular dilation and/or cystic periventricular leukomalacia and/or >6 punctate white matter lesions and/or focal cerebellar hemorrhage and/or brain malformations.

In a subgroup analysis, the effect of anthropometric growth during hospitalization on neurodevelopmental outcomes was evaluated excluding the subjects who had developed SBL. A subgroup analysis was also performed considering small for gestational age [SGA, i.e., with a birth weight <10th centile for gestational age according to the INeS Charts (22)] patients.

All tests were two-tailed. A *p*-value <0.05 was considered statistically significant. The statistical analyses were performed using R software (Foundation for Statistical Computing, Wien, Austria).

## 3. Results

### 3.1. Basic characteristics of study population

**Figure 1:**
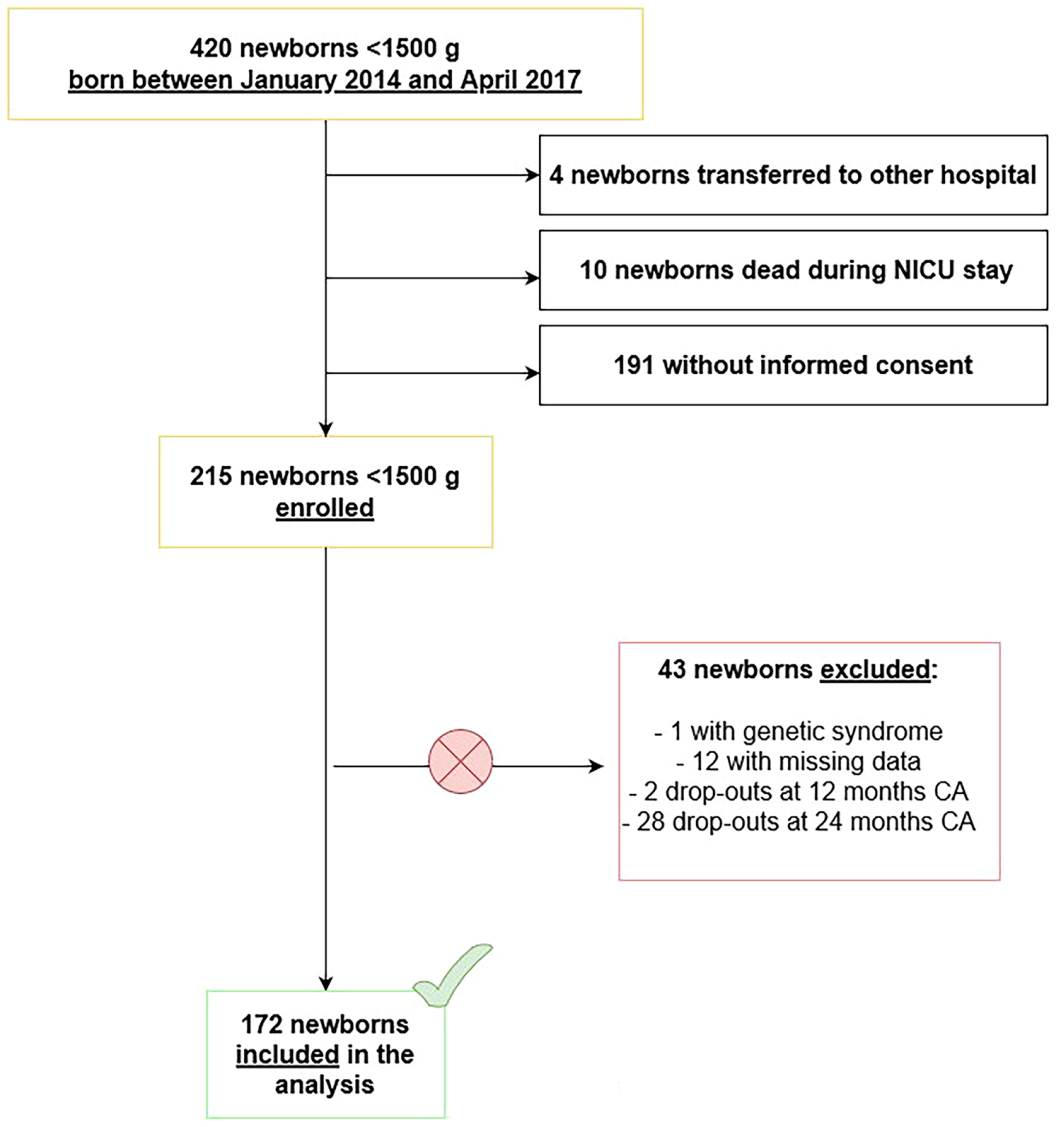
Patients’ flow through the study.

Patients' flow through the study is represented in Figure 1.

The basic socio-demographic characteristics of the study population are summarized in Table 1.

**Table 1:** Clinical and socio-demographic characteristics of the study population.

Variable	Value
Neonatal characteristics (*N* = 172)	
Gestational age (weeks), mean (SD)	29.3 (2.2)
<28, *N* (%)	36 (20.9)
28^+0^–31^+6^, *N* (%)	117 (68.0)
32^+0^–33^+6^, *N* (%)	13 (7.6)
34^+0^–36^+6^, *N* (%)	6 (3.5)
Females, *N* (%)	91 (52.9)
Caesarean delivery, *N* (%)	154 (89.5)
Twins, *N* (%)	85 (49.4)
Apgar 1′/5′, median (IQR)	7 [6–8]/8 [8–9]
Birth weight (g), mean (SD)	1,116.7 (254.5)
VLBW, *N* (%)	108 (62.8)
ELBW, *N* (%)	64 (37.2)
SGA, *N* (%)	41 (23.8)
Length (cm), mean (SD)	37.1 (3.2)
Head circumference (cm), mean (SD)	26.2 (2.2)
NICU los (days), median (IQR)	61 [47–84]
Exclusive breastfeeding, *N* (%)	39 (22.7)
Mixed feeding, *N* (%)	48 (27.9)
Exclusive formula feeding, *N* (%)	85 (49.4)
Maternal characteristics (*N* = 172)	
Age 30–40 years, *N* (%)	109 (63.7)
European Nationality, *N* (%)	133 (77.3)
University degree, *N* (%)	74 (43.3)
Corticosteroid prohylaxis, *N* (%)	166 (96.5)
Paternal characteristics (*N* = 172)	
Age 30–40 years, *N* (%)	82 (50)
European Nationality, *N* (%)	138 (80.2)
University degree, *N* (%)	59 (36)

ELBW, extremely low birth weight; IQR, inter-quartile range; los, length of stay; NICU, neonatal intensive care unit; SD, standard deviation; SGA, small for gestational age; VLBW, very low birth weight.

The study population included 172 (81 males, 47.1%) subjects with an average gestational age of 29 weeks and an average birth weight of 1,117 g. Among subjects included in the analysis, 108 were VLBW (i.e., with a birth weight <1,500 g), corresponding to 62.8% of the population studied, and 64 were Extremely Low Birth weight (ELBW, i.e., with a birth weight <1,000 g). Mean length and head circumference at birth were 37 cm and 26 cm, respectively. The 23.8% of the subjects included in the study were SGA.

Mean hospitalization in the NICU lasted 61 days, and 22.7% of infants were exclusively breastfed at discharge.

**Figure 2:**
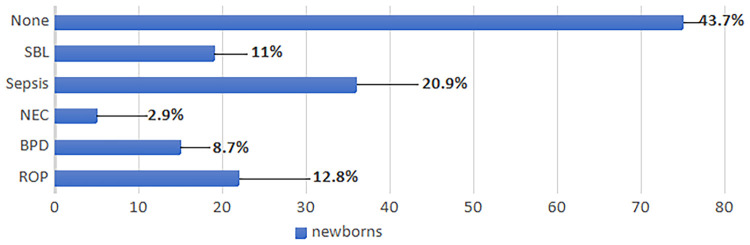
Comorbidities of the study population. BPD, bronchopulmonary dysplasia; NEC, necrotizing enterocolitis; ROP, retinopathy of prematurity; SBL, severe brain lesions.

Figure 2 shows the prevalence of the reported comorbidities in the study population.

### 3.2. Anthropometric growth

The auxological parameters of the study population, expressed as z-scores, are summarized in Table 2. In terms of weight, the study population remained below the expected average throughout hospitalization, both at weight recovery and at discharge. At 12 and 24 months CA the population recovered the weight, even though the mean remained below 0. Likewise, length and head circumference improved at 12 and 24 months CA.

**Table 2:** Auxological parameters of the study population throughout the study period.

Parameter	Mean z-score (SD)				
	Birth	Weight Recovery	Discharge	12 months	24 months
Weight	−0.50 (1.05)	−0.79 (1.58)	−1.09 (1.32)	−0.48 (1.14)	−0.52 (1.33)
Length	−0.60 (1.11)		−1.62 (1.78)	−0.83 (1.54)	−0.57 (1.19)
Head circumference	−0.45 (1.09)		−1.15 (1.56)	0.02 (1.61)	0.23 (1.55)

According to extra-uterine growth restriction (EUGR) longitudinal definitions, we found that 45 (26.2%) patients had a loss of weight z-score >1 SDS from birth to discharge, 33 (19.2%) patients had a loss of weight z-score >1 SDS from weight recovery to discharge, 77 (44.8%) patients had a loss of length z-score >1 SDS from birth to discharge, 68 (39.5%) patients had a loss of head circumference z-score >1 SDS from birth to discharge.

### 3.3. Neurodevelopmental outcomes

**Figure 3:**
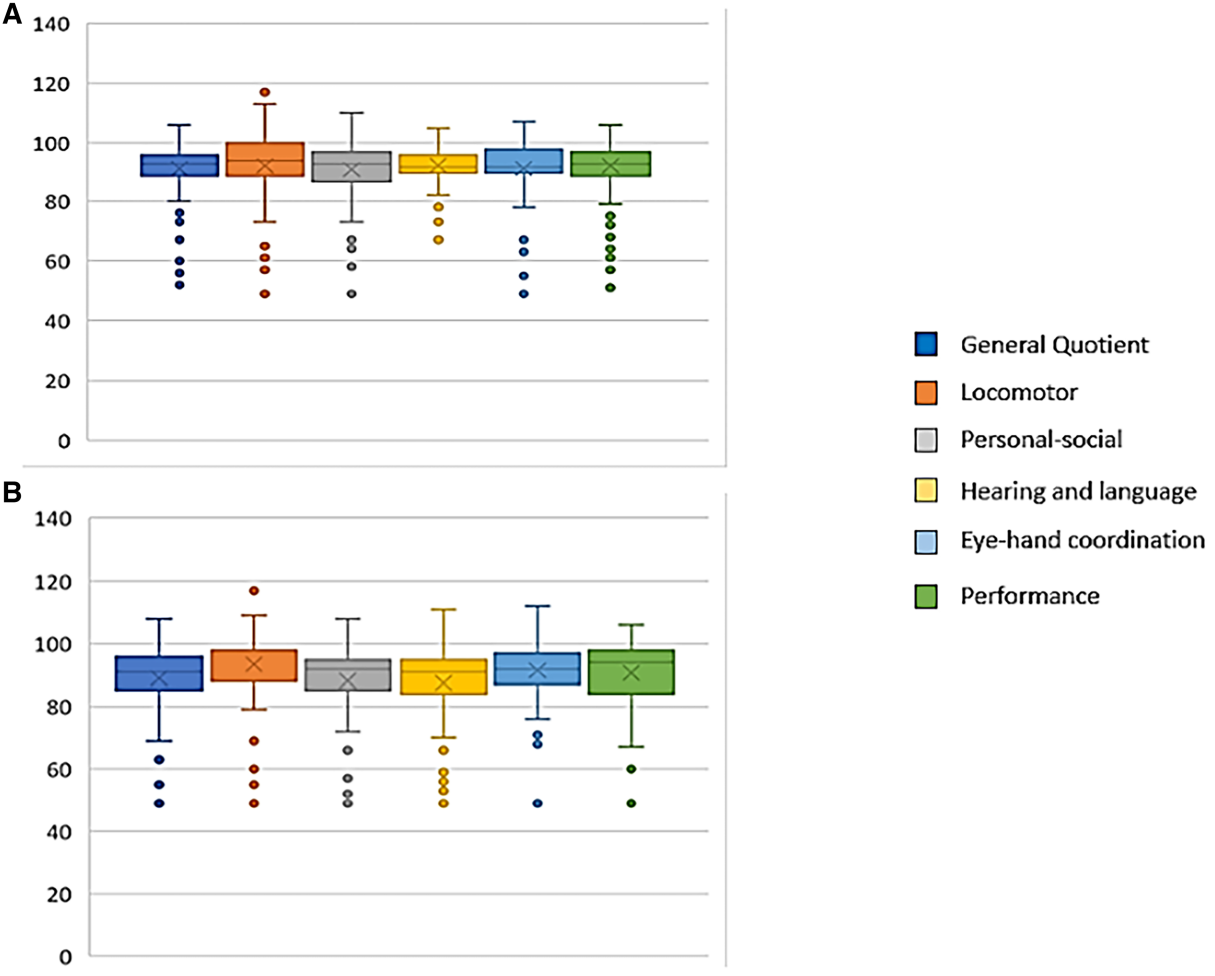
Distribution of GMDS-ER scores of the study population. **A**: GMDS-ER scores at 12 months CA; **B**: GMDS-ER scores at 24 months CA.

Figure 3 shows the score distribution of the General Quotient and the 5 subscales of the GMDS-ER.

All infants included in the analysis underwent GMDS-ER. Only one infant resulted unable to perform the GMDS-ER, due to severe cerebral palsy, and was therefore scored <49 in General Quotient and all subscales. However, because of missing data, said patient was not included in the final analysis.

At 12 months CA mean General Quotient was 91.4, with a mean score of 92.4 in subscale A—locomotor, 91.1 in subscale B—personal-social, 92.4 in subscale C—hearing and language, 91.5 in subscale D—eye-hand coordination, and 92.3 in subscale E—performance.

At 24 months CA mean General Quotient was 89.0, with a mean score of 93.4, 88.3, 87.5, 91.4 and 90.7 in subscales A—E, respectively.

### 3.4. Association between auxological parameters and neurodevelopmental outcome

In the studied population a significant association was found at 12 months CA between weight and length Δz-score from birth to discharge and GMDS-ER subscale C scores (a gain of 0.82 and 0.65 for each Δz-score point, respectively) (Table 3). Conversely, no significant association was found considering the weight increase from recovery of birth weight (data not shown). The increase in head circumference from birth to discharge showed an association with subscales B and C, although not reaching statistical significance (*p* = 0.08 and *p* = 0.07, respectively).

**Table 3:** Results of the multivariate linear regression model showing the association between auxological growth during NICU stay and neurodevelopmental outcome assessed through the GMDS-ER at 12 and 24 months CA in the study population.

	12 months			24 months		
	Estimate	95% CI	*p*-value	Estimate	95% CI	*p*-value
	*Weight* Δ*z-score*					
General Quotient	0,359	−0.734; 1.453	0,517	0,964	−0.676; 2.605	0,247
A—Locomotor	−0,058	−1.732; 1.617	0,946	0,723	−0.926; 2.372	0,388
B—Personal-Social	0,409	−0.867; 1.684	0,528	0,784	−0.902; 2.47	0,360
C—Hearing—Language	0,819	0.037; 1.601	**0,040**	1,256	−0.714; 3.226	0,210
D—Eye—Hand Coordination	0,100	−1.163; 1.362	0,876	0,647	−0.866; 2.16	0,400
E—Performance	−0,083	−1.308; 1.142	0,894	0,330	−1.235; 1.894	0,678
	*Length* Δ*z-score*					
General Quotient	0,159	−0.511; 0.829	0,640	0,485	−0.518; 1.488	0,341
A—Locomotor	−0,180	−1.204; 0.844	0,729	0,133	−0.878; 1.144	0,796
B—Personal-Social	0,308	−0.47; 1.087	0,435	0,380	−0.652; 1.413	0,468
C—Hearing—Language	0,651	0.177; 1.125	**0,007**	1,012	−0.186; 2.21	0,097
D—Eye—Hand Coordination	0,190	−0.579; 0.96	0,626	0,213	−0.714; 1.139	0,651
E—Performance	−0,445	−1.192; 0.301	0,240	0,002	−0.954; 0.959	0,996
	*Head circumference Δz-score*					
General Quotient	0,492	−0.285; 1.27	0,213	1,614	0.42; 2.808	**0,008**
A—Locomotor	0,081	−1.128; 1.29	0,895	0,953	−0.26; 2.167	0,123
B—Personal-Social	0,807	−0.111; 1.724	0,084	1,183	−0.052; 2.418	0,060
C—Hearing—Language	0,511	−0.046; 1.068	0,072	2,089	0.657; 3.52	**0,005**
D—Eye—Hand Coordination	0,655	−0.266; 1.576	0,162	1,431	0.337; 2.525	**0,011**
E—Performance	0,289	−0.606; 1.185	0,524	1,016	−0.123; 2.156	0,080

Estimates, confidence intervals and *p*-values from multivariate regression model adjusted for mode of feeding, comorbidity score and gestational age.

Bold values indicate statistical significance (*p* < 0.05).

At 24 months CA an increase in head circumference of 1 unit of Δz-score was found to be associated with an average increase in the General Quotient of 1.6 points. A statistically significant association with subscales C and D was also found, with a gain of 2 and 1.4 points, respectively, as well as a non-statistically significant association with subscales B (*p* = 0.06) and E (*p* = 0.08). As for the increase in length, the greatest effect was observed on subscale C (1 point for each unitarian variation of Δz-score, *p* = 0.09). On the other hand, no relationship with the neurodevelopmental outcome at 24 months could be found with regards to weight gain.

### 3.5. Sub-group analysis: patients without SBL

Within the study population, 153 newborns did not develop SBL. These newborns had a mean gestational age of 29 weeks, a mean birth weight of 1,126 g, a mean length at birth of 37 cm and a mean head circumference at birth of 26 cm. At discharge, weight, length and head circumference mean z-scores were −1.12, −1.65 and −1.10, respectively.

The association between auxological parameters of the patients without SBL and neurodevelopmental outcome at 12 and 24 months CA is shown in Table 4.

**Table 4:** Results of the multivariate linear regression model showing the association between auxological growth during NICU stay and neurodevelopmental outcome assessed through the GMDS-ER at 12 and 24 months CA in the population without SBL.

	12 mMonths			24 months		
	Estimate	95% CI	*p*-value	Estimate	95% CI	*p*-value
	*Weight Δz-score*					
General Quotient	0.072	−0.984; 1.128	0.893	1.061	−0.602; 2.724	0.209
A—Locomotor	−0.444	−2.026; 1.138	0.580	0.082	−1.329; 1.493	0.909
B—Personal-Social	0.171	−1.155; 1.497	0.799	1.082	−0.676; 2.840	0.226
C—Hearing—Language	0.666	−0.103; 1.436	0.089	1.789	−0.218; 3.797	0.080
D—Eye—Hand Coordination	−0.058	−1.370; 1.255	0.931	0.717	−0.757; 2.191	0.338
E—Performance	−0.172	−1.409; 1.064	0.783	0.256	−1.235; 1.746	0.735
	*Length Δz-score*					
General Quotient	0.190	−0.445; 0.825	0.555	0.687	−0.312; 1.686	0.176
A—Locomotor	−0.207	−1.159; 0.745	0.668	0.288	−0.561; 1.137	0.504
B—Personal-Social	0.343	−0.451; 1.138	0.395	0.561	−0.499; 1.621	0.297
C—Hearing—Language	0.661	0.207; 1.116	**0** **.** **005**	1.348	0.150; 2.546	**0** **.** **028**
D—Eye—Hand Coordination	0.357	−0.428; 1.142	0.371	0.335	−0.554; 1.223	0.458
E—Performance	−0.285	−1.028; 0.458	0.450	0.081	−0.816; 0.977	0.859
	*Head circumference Δz-score*					
General Quotient	0.549	−0.247; 1.346	0.175	1.714	0.442; 2.986	**0** **.** **009**
A—Locomotor	0.115	−1.076; 1.307	0.848	0.908	−0.179; 1.995	0.101
B—Personal-Social	0.800	−0.196; 1.796	0.115	1.254	−0.107; 2.616	0.071
C—Hearing—Language	0.620	0.040; 1.200	**0** **.** **036**	2.321	0.789; 3.853	**0** **.** **003**
D—Eye—Hand Coordination	0.731	−0.257; 1.719	0.146	1.421	0.294; 2.548	**0** **.** **014**
E—Performance	0.395	−0.542; 1.333	0.406	0.997	−0.152; 2.147	0.089

Estimates, confidence intervals and *p*-values from multivariate regression model adjusted for mode of feeding and comorbidity score.

Bold values indicate statistical significance (*p* < 0.05).

The increase in weight from birth to discharge showed a non-statistically significant association with subscale C at 24 months CA (*p* = 0.08), and no relationship was found between the weight increase from recovery of birth weight (data not shown).

The positive effect of the increase of head circumference Δz-score throughout hospitalization on neurodevelopmental outcomes seen in the total study population was confirmed at 24 months CA in the sub-group analysis.

### 3.6. Sub-group analysis: SGA patients

Within the study population, 41 (23.8%) newborns were born SGA. The association between auxological parameters of SGA patients and neurodevelopmental outcome at 12 and 24 months CA is shown in Supplementary Table S1.

The increase in weight from birth to discharge showed an association with subscale C at 12 months CA, although not reaching statistical significance (*p* = 0.07). No association was found at 24 months CA.

The increase in length from birth to discharge showed an association with subscale C at 24 months CA. but not at 12 months CA.

For each unitarian variation of head circumference Δz-score a 4.2-point increase in subscale C scores at 24 months CA was observed. No association with neurodevelopmental outcome at 12 months CA was observed.

## Discussion

The results of the present study show a positive association between growth of head circumference during hospital stay and neurodevelopmental outcome at 24 months CA, measured through the GMDS-ER scale. This association is evident not only with regards to the General Quotient but also when considering the specific subscales investigating the hearing and language and eye-hand coordination domains. It is important to underline that such positive association was found not only in the whole cohort of premature infants but also in those who did not develop SBL. This data highlights the importance of monitoring head circumference already during the NICU stay in all newborns, regardless of the presence of other known risk factors for adverse neurodevelopmental outcomes.

Previous studies (11, 23–26) have documented the positive impact of head growth during hospitalization on neurodevelopmental outcomes. In particular, De Rose et al. (26) showed that a loss of z-score >1 SDS in weight and head circumference, calculated from when physiological weight loss is over and identified as soon as possible rather than at discharge, better predicts neurodevelopmental outcomes of very preterm infants. Interestingly, these Authors identified significant differences in subscales A, B, D and E, but not in subscale C. Moreover, several studies in the Literature have pointed out that head circumference represents an excellent indicator of brain volume assessed through MRI, thus suggesting that its accurate measurement over time can be used as an easily assessable proxy of the global brain volume during the first years of life (27, 28).

In our study, a greater growth in length during hospitalization was found to be associated with a higher score at the hearing and language subscale. This result could be at least partially explained by the fact that linear growth reflects lean mass accretion which, in turn, has been associated with a better neurodevelopmental outcome (29). In line with our results, Ramel et al. (30) reported for each unitarian increase in length z-score in the first 4 and 12 months CA a gain of 4 points in 24-month neurocognitive scores assessed through the Bayley scale of infant development. Interestingly, when controlling for length and head circumference, the association between weight z-scores at any time point and 24-month neurodevelopmental outcomes was lost.

The hearing and language subscale evaluates hearing, expressive and receptive language. Developmental language disorders are common in preterm children (31) and appear to be independent of the presence of significant abnormalities in brain morphology, IQ scores and neurological examination (32). In line with these findings, a recent Italian study (33) documented in a cohort of ELBW infants a peculiar developmental profile assessed through the GMDS-ER showing a selective deficit in the language domain. A possible role of early post-natal nutritional deficits could be implicated in the recently reported relative sparing of motor system compared to speech and cognitive scores when assessing the effects of growth retardation on neurodevelopmental outcomes (30, 33). Indeed, brain development and maturation is a dynamic process, occurring in different overlapping time-periods within different brain areas (34). Contrary to the motor cortex, which begins its rapid development after 40 weeks post-menstrual age, the hippocampus, that presides declarative memory, implicated in supporting lexical knowledge and retrieving semantic knowledge (35), is among the most rapidly developing regions during late fetal and early neonatal life, thus potentially being particularly susceptible to early nutritional deficits (30).

In our total study population, we found an association between weight gain during NICU stay and subscale C at 12 months CA, whereas no relationship was found with neurodevelopmental outcomes at 24 months CA. When considering only the newborns who did not develop SBL or those who were born SGA no association was found between weight gain and neurodevelopmental outcome at 12 nor 24 months CA.

Our results appear to be in contrast with the current Literature. Latal-Hajnal et al. documented an association between poor post-natal growth and neurodevelopmental impairment in a cohort of VLBW infants (defined as having a birth weight <1,250 g) (36). However, in their study the Authors assessed the enrolled infants' anthropometric parameters (weight, length and head circumference) at 9 and 24 months, whereas no data is reported on growth during NICU stay. Conversely, in a cohort of premature infants born <33 weeks gestational age, Belfort et al. observed only a modest association between a greater weight gain from week 1 of life to term and higher cognitive and motor scores at 18 months CA. A smaller impact on motor outcomes was documented considering weight gain from term to 4 months and no effect on neurodevelopmental outcomes at 18 months was observed for weight gain from 4 to 12 months (37). Finally, Ehrenkranz et al. reported that a faster in-hospital weight gain velocity was associated with better neurodevelopmental outcomes assessed through the Bayley scale at 18 to 22 months CA in a cohort of ELBW infants (11). In our study we did not consider weight gain velocity but only assessed the absolute weight gain from birth to discharge, in terms of Δz-score.

More in line with our findings, Maiocco et al. (25) showed in a cohort of infants born <30 weeks gestational age that, even though weight cross-sectional EUGR (i.e., weight at discharge) was associated with minor neurodevelopmental impairment at 24 months CA at univariate analysis, such association lost its statistical significance when correcting for confounding factors. Furthermore, no association with neurodevelopmental outcomes at 24 months CA was found considering the longitudinal and post-loss definitions of EUGR. Likewise, a recent review article by Fenton et al. (38) concluded that EUGR is not predictive of neurodevelopmental outcomes.

In our study we did not consider EUGR *per se*, deeming the heterogeneity of existing definitions a limit to the interpretation of results and their comparability between studies. Indeed, EUGR is variously defined in the Literature and can be measured either at a given time (cross-sectional EUGR) or between two time points (longitudinal EUGR) (25, 38, 39). Such variability inevitably affects the reported prevalence of EUGR in different studies, which also varies according to the growth charts used, as well as its impact on infants' outcomes (26). We thus decided to only consider the absolute value of the measurements performed and their relative z-scores.

Our results could depend on the fact that weight, albeit easier to measure than other auxological parameters, is a rather crude indicator of growth (25), especially if body composition is not evaluated. Indeed, it has been nicely documented how in preterm infants born <30 weeks gestational age weight gain during NICU stay is weakly associated with higher absolute lean mass in infancy. However, such association does not persist after correction for length (40).

In clinical practice the assessment of body composition is often complicated by the clinical instability of the hospitalized newborns and the limited availability of adequate methods, such as air plethysmography or dual-energy x-ray absorptiometry (DEXA). The longitudinal evaluation of more readily assessable anthropometric parameters, like length and head circumference, can therefore represent a valid proxy of the quality of growth.

Further studies are needed to clarify the relative importance of weight gain during NICU stay in terms of long-term neurodevelopmental outcomes of preterm VLBW infants.

Major strengths of our study are the inclusion of a relatively large cohort of premature VLBW infants, who received standardized nutritional care and were followed longitudinally up to 24 months CA with a low drop-out rate (0.9% at 12 months CA and 13% at 24 months CA) (41). The use of the Comorbidity score allowed us to correct our model for known risk factors for neurodevelopmental impairment. Moreover, by performing a subgroup analysis we demonstrated consistency of our results, that appear to be independent of the presence of SBL. This underlines the importance of monitoring growth during NICU stay in all preterm VLBW newborns, regardless of the presence of comorbidities, even neurological. However, our study has the intrinsic limitations of a monocentric design. The other main limitation of our study is the lack of a body composition analysis correlate, which might have supported the interpretation of our results. To avoid the potential limitation of a wide range of gestational ages, we corrected our model also for gestational age. Finally, we acknowledge the apparent limited effect reported in our study on neurodevelopmental outcomes (e.g., 1.6-point increase in general quotient or 2-point increase in subscale C scores for each unitarian variation of head circumference Δz-score). However, we believe that demonstrating even small improvements in neurodevelopmental outcomes underlines the importance of monitoring growth already during the NICU stay and not just post-discharge, in order to timely identify newborns at higher risk for neurodevelopmental impairment and tailor nutritional and non-nutritional strategies aimed at promoting their growth accordingly. Comparison of the time windows (in-hospital vs. post-discharge) for predicting neurodevelopmental outcomes could also have been interesting, although beyond the purposes of the present study.

## Conclusions

In our cohort, the increase in length and head circumference during NICU stay was related to a more favorable neurodevelopmental outcome at 24 months CA, especially in the hearing and language domain. Conversely, weight gain was not associated with neurodevelopmental outcomes at 24 months CA in our cohort. We suggest to longitudinally evaluate auxological parameters (especially length and head circumference) throughout hospitalization, using suitable tools and appropriate curves for the study population. This can contribute to the identification of subjects at risk for adverse neurodevelopmental outcome in the first years of life, allowing for timely nutritional interventions that favor growth and neurodevelopment.

## Data Availability

The raw data supporting the conclusions of this article will be made available by the authors, without undue reservation.
