# Supplementary material for: In-hospital growth and long-term neurodevelopmental outcomes of very low birth weight infants
Source: Front Pediatr. 2023 May 11;11:1180068. doi: 10.3389/fped.2023.1180068 (PMC10211263; doi:10.3389/fped.2023.1180068)
Supplement: Supplementary file 1 [file Table1.docx]

|  |  |  |  |  |  |  |
| --- | --- | --- | --- | --- | --- | --- |
|  | **12 Months** | | | **24 Months** | | |
|  | **Estimate** | **95% CI** | **p value** | **Estimate** | **95% CI** | **p value** |
|  | ***Weight Δz-score*** | | | | | |
| **General Quotient** | 0,930 | -1.998; 3.857 | 0,523 | 1,981 | -1.429; 5.391 | 0,246 |
| **A - Locomotor** | 0,298 | -3.155; 3.752 | 0,862 | 1,348 | -1.877; 4.573 | 0,402 |
| **B - Personal-Social** | 0,821 | -2.451; 4.094 | 0,614 | 1,826 | -1.701; 5.354 | 0,300 |
| **C - Hearing - Language** | 1,608 | -0.141; 3.356 | 0,070 | 2,858 | -1.338; 7.054 | 0,175 |
| **D - Eye – Hand Coordination** | 0,672 | -2.599; 3.942 | 0,679 | 1,620 | -1.701; 4.942 | 0,329 |
| **E - Performance** | 0,069 | -3.185; 3.324 | 0,966 | 1,146 | -1.654; 3.946 | 0,412 |
|  | ***Length Δz-score*** | | | | | |
| **General Quotient** | 0,259 | -1.544; 2.062 | 0,773 | 1,461 | -0.611; 3.533 | 0,161 |
| **A - Locomotor** | 0,297 | -1.819; 2.413 | 0,777 | 0,606 | -1.38; 2.593 | 0,540 |
| **B - Personal-Social** | 0,246 | -1.766; 2.258 | 0,806 | 1,284 | -0.868; 3.436 | 0,234 |
| **C - Hearing - Language** | 0,808 | -0.281; 1.897 | 0,141 | 2,569 | 0.079; 5.059 | **0,044** |
| **D - Eye – Hand Coordination** | 0,287 | -1.72; 2.295 | 0,773 | 0,954 | -1.084; 2.993 | 0,348 |
| **E - Performance** | -0,420 | -2.41; 1.57 | 0,671 | 0,330 | -1.399; 2.06 | 0,701 |
|  | ***Head circumference Δz-score*** | | | | | |
| **General Quotient** | 0,295 | -2.283; 2.873 | 0,817 | 2,532 | -0.393; 5.457 | 0,088 |
| **A - Locomotor** | -0,659 | -3.739; 2.42 | 0,666 | 1,266 | -1.331; 3.864 | 0,329 |
| **B - Personal-Social** | 0,702 | -2.254; 3.658 | 0,632 | 2,522 | -0.489; 5.533 | 0,098 |
| **C - Hearing - Language** | 0,999 | -0.515; 2.514 | 0,189 | 4,253 | 0.691; 7.815 | **0,021** |
| **D - Eye – Hand Coordination** | 0,833 | -2.189; 3.854 | 0,579 | 2,120 | -0.703; 4.943 | 0,136 |
| **E - Performance** | -0,140 | -3.125; 2.845 | 0,925 | 0,916 | -1.462; 3.293 | 0,439 |
|  |  |  |  |  |  |  |
|  |  |  |  |  |  |  |

**Supplementary Table 1. Results of the multivariate linear regression model showing the association between auxological growth during NICU stay and neurodevelopmental outcome assessed through the GMDS-ER at 12 and 24 months CA in patients born SGA**.

*Estimates, confidence intervals and p values from multivariate regression model adjusted for mode of feeding and comorbidity score.*
